# Colorectal cancer and gut viruses: a visualized analysis based on CiteSpace knowledge graph

**DOI:** 10.3389/fmicb.2023.1239818

**Published:** 2023-10-18

**Authors:** Chu Jian, Zhuang Jing, Wu Yinhang, Duan Jinlong, Pan Yuefen, Qi Quan, Han Shuwen

**Affiliations:** ^1^Fifth School of Clinical Medicine of Zhejiang Chinese Medical University (Huzhou Central Hospital), Huzhou, China; ^2^Huzhou Central Hospital, Affiliated Central Hospital Huzhou University, Huzhou, China; ^3^Key Laboratory of Multiomics Research and Clinical Transformation of Digestive Cancer, Huzhou, China; ^4^Huzhou Hospital of Traditional Chinese Medicine, Huzhou, China

**Keywords:** colorectal cancer, gut microbiota, bibliometric, virus, bacteriophage

## Abstract

**Background:**

Gut microbiome is a complex community of microbes present in the human gut and plays an important role in the occurrence and progression of colorectal cancer (CRC). However, the relationship between virus and CRC has not been fully understood.

**Objective:**

To explore the hot spots and research trends in the field of CRC and virus.

**Methods:**

By using the bibliometric analysis tool CiteSpace and based on the articles of the Web of Science Core Collection (WoSCC) database, the country, institution, highly cited literature, keywords and so on were visually analyzed.

**Results:**

A total of 356 research articles on CRC from 2001 to 2023 were thoroughly analyzed. The USA and China have made the largest contribution in the field of virus and CRC. The Helmholtz Association published the most papers. There were relatively few cooperations among institutions from different countries. The results of keyword cluster analysis proved that the literature on the relationship between human cytomegalovirus (CMV) and CRC was the most widely studied aspect in this field. “Gut microbiota,” “inflammatory bowel disease,” “hepatitis b virus,” and “human papillomavirus infection” are the current research hotspots; “oncolytic virus,” “apoptosis,” and “gut microbiome” are the recent research frontiers and should be paid closer attention.

**Conclusion:**

By using CiteSpace bibliometric software, the visual analysis reflected the research trends and hot topics of virus and CRC. In addition, the prevalence and mechanism of specific virus on CRC were also reviewed, which provides valuable references for future CRC research.

## 1. Introduction

Colorectal cancer (CRC) is the third most common cancer in males and the second most common cancer in females worldwide, accounting for about 10% of all new cancer cases worldwide. Nearly two million cases of CRC occur each year, which causes one million deaths, thus making it one of the major global disease burdens (Ferlay et al., 2015). According to statistics, there were 1.9 million new cases of CRC in 2020, and it is expected to reach 2.5 million in 2035 (Sung et al., 2021). Every person has an approximately 4% of lifetime risk of developing CRC, but some factors can increase this likelihood (Simon, 2016; Ma et al., 2018). Several environmental factors have been linked to CRC, such as age [≥ 50 years old (Levin et al., 2008)], personal medical history [ulcerative colitis (Eaden et al., 2001) and Crohn’s disease (Canavan et al., 2006)], poor lifestyle (sedentary), unhealthy diet, and gut microenvironment.

Growing evidence over the past 20 years has shown how crucial gut microbiota is for host’s metabolic health and immunological homeostasis. The predominant microorganisms in the gut microecological environment include bacteria, fungi, viruses, and associated metabolites. The gut microbiota related to CRC was different compared with that of healthy individuals as controls, with a higher species richness, lower abundance of potentially protective taxa (for example, Roseburia), and increased abundance of procarcinogenic taxa (such as Bacteroides, Escherichia, Fusobacterium, and Porphyromonas) (Castellarin et al., 2012; Feng et al., 2015; Yu et al., 2017). Past studies on CRC and gut microbes focused on gut bacteria. Intestinal microorganisms also include viruses, and many studies have found that gut viruses are currently related to CRC. For example, Epstein–Barr virus (EBV) and human papillomavirus (HPV), cytomegalovirus (CMV or human herpesvirus type 5), John Cunningham virus (JCV) (Selgrad et al., 2008; Costa et al., 2018; Mirzaei et al., 2018), cytomegalovirus (CMV) (Marongiu and Allgayer, 2022), and etc. have been consistently reported to be prevalent in CRC.

EBV-encoded miRNAs (EBV-miRs) play an indispensable role in the pathogenesis and progression of EBV-associated tumors. Recent studies have proved that EBV-miR-BART18-3p contributed to and promoted CRC metastasis during EBV injection via an altered lipogenesis pathway (Tsao et al., 2015; Elgui de Oliveira et al., 2016). Also, several studies have highlighted the presence of high-risk HPV (HPVs-16, 18, 31, 33, and 35) in CRC (Buyru et al., 2006; Salepci et al., 2009; Yavuzer et al., 2011; Ghabreau et al., 2012). In this regard, Qiu et al. (2020) performed a gene expression analysis and found four upregulated and differentially expressed genes in HPV-positive CRC samples compared with HPV-negative tissues. These genes coded proteins, namely WNT-5A, c-myc, matrix metalloproteinase 7 (MMP-7), and AXIN2, which have been previously implicated in CRC pathogenesis (Qiu et al., 2020). Li et al. (2015) reported a correlation between HPV infection and worse clinical stages of CRC. Karbasi et al. (2015) found a significantly lower expression of the pro-apoptotic genes FAS and DR5 in HPV-positive CRC samples compared with normal tissue. Studies have also determined that there may be an association between HPV infection and K-Ras (Buyru et al., 2006), p53 mutations (Sayhan et al., 2001; Buyru et al., 2003). JCV has been reported to have the potential to promote colon carcinogenesis in a variety of ways. The genome of JCV encodes a transforming protein, T-antigen, which is thought to be involved in the oncogenic properties of the virus and can interact with p53 and pRB tumor suppressor proteins as well as other major signaling pathways (Hampras et al., 2014). Human cytomegalovirus (CMV) has been shown to preferentially infect CRC lesions over normal healthy tissue (Damin et al., 2013; Dimberg et al., 2013), which may be associated with a poor prognosis in patients with CRC (Chen et al., 2014, 2015, 2016). This phenomenon may be associated with proliferation and progression of CRC cells, where the expression of TLR2, TLR4, NF-κB, and TNF-α is higher than in control tissues in CMV-infected CRC samples (Li et al., 2015), and the expression levels of Bcl-2, cox-2, and Wnt/β-catenin are elevated in cancer cell lines (Harkins et al., 2002; Teo et al., 2017).

Bacteriophages are viruses that attack bacteria and genetic materials that confer biological traits on the host bacteria. Bacteriophages are the most common and widely distributed group of viruses (Waldor, 1998). Recent work has shown differences in the prevalence of bacteriophages between the healthy and inflamed intestine (Zuo et al., 2019). Nakatsu et al. (2018) found that gut bacteriophage community is significantly increased in patients with CRC. In addition, phage-induced bacteriolysis releases cellular debris into the microenvironment, which can induce inflammation.

Bibliometric analysis, a statistical method based on public literature databases (e.g., Web of Science), is a useful tool for statistically and qualitatively assessing trends in research work. Keywords that appear frequently in the included articles and hot words that have emerged in recent years were analyzed to provide supporting evidence for future trends (Wang et al., 2021). In recent years, many publications on gut viruses and CRC have been published, while a systematic study on the association between gut viruses and CRC through bibliometric analysis has not yet been explored. This paper analyzed the co-occurrence of relevant literature on the relationship between gut virus and CRC through CiteSpace software, and drawn a knowledge map to visualize the research dynamics, change patterns and development processes, to identify the academic hotspots of research in both fields, with a view to providing new ideas and references for future relevant researches.

## 2. Methods

### 2.1. Data source and retrieval strategy

The Web of Science (WOS) core database from Clarivate Analytics was deemed as the best for bibliometric analysis (Aggarwal et al., 2016; Ke et al., 2020), so it was used as the data source. The WOS core database was searched on May 6, 2023, for all articles related to the relationship between CRC and gut viruses. All relevant publications are collected primarily on the basis of titles (T1) and abstracts (AB), using the following search formula: #1: (AB = ((Rectal Neoplasm*) OR (Rectal Tumor*) OR (Rectal Cancer*) OR (Rectum Neoplasm*) OR (Rectum Cancer*) OR (Cancer of the Rectum) OR (Cancer of Rectum) OR (Colorectal Neoplasm*) OR (Colorectal Tumor*) OR (Colorectal Cancer*) OR (Colorectal Carcinoma*) OR (Colonic Neoplasm*) OR (Colon Neoplasm*) OR (Cancer of Colon) OR (Colon Cancer*) OR (Cancer of the Colon) OR (Colonic Cancer*))) OR (TI = ((Rectal Neoplasm*) OR (Rectal Tumor*) OR (Rectal Cancer*) OR (Rectum Neoplasm*) OR (Rectum Cancer*) OR (Cancer of the Rectum) OR (Cancer of Rectum) OR (Colorectal Neoplasm*) OR (Colorectal Tumor*) OR (Colorectal Cancer*) OR (Colorectal Carcinoma*) OR (Colonic Neoplasm*) OR (Colon Neoplasm*) OR (Cancer of Colon) OR (Colon Cancer*) OR (Cancer of the Colon) OR (Colonic Cancer*))); #2: (AB = (virus* OR virology* OR HPV OR EBV OR CMV OR JCV)) OR (TI = (virus* OR virology* OR HPV OR EBV OR CMV OR JCV)); Final dataset: #1 AND #2. The search articles were published in English from 2001 (1 January 2001) to 2023 (5 May 2023).

**Figure 1:**
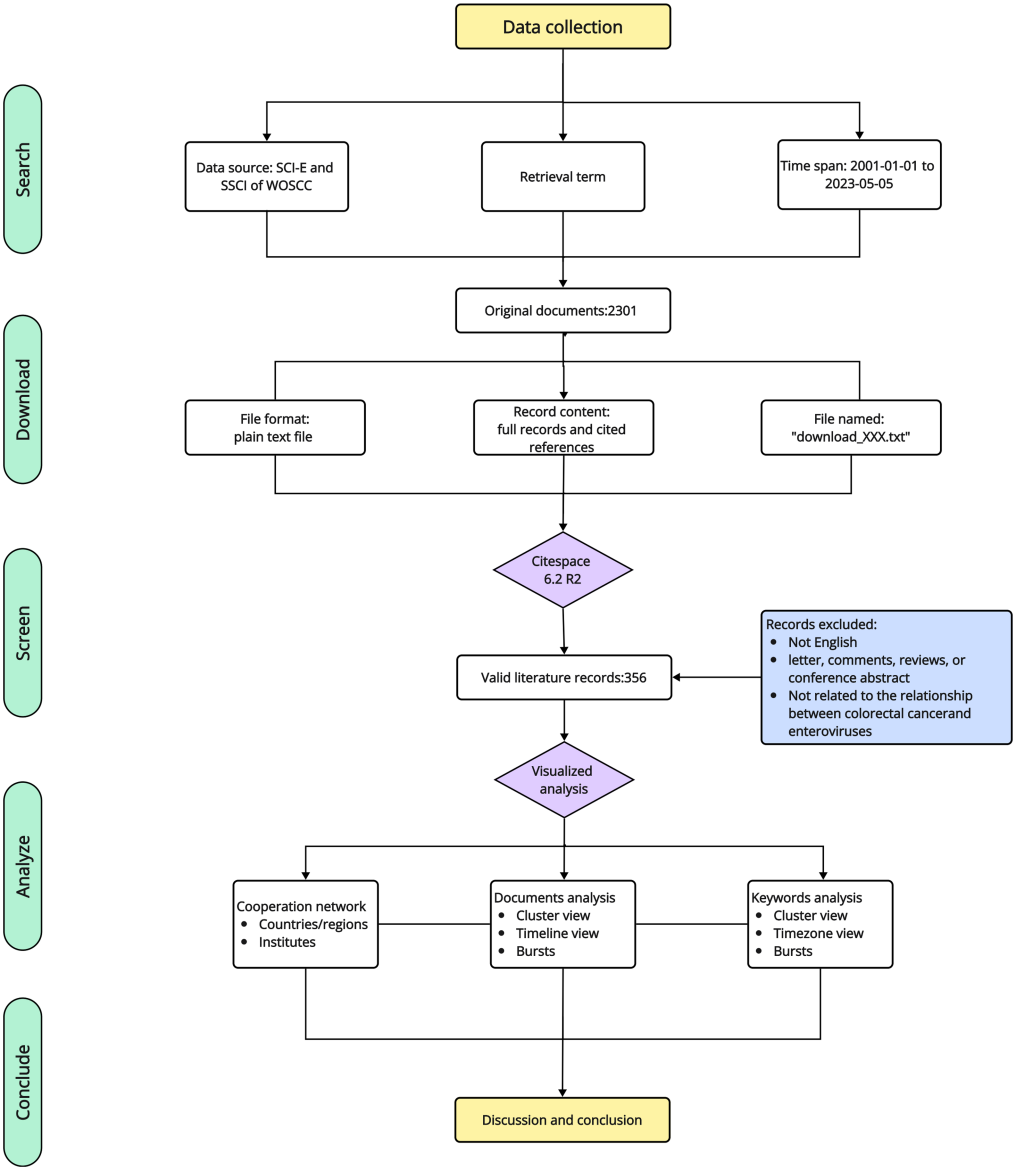
Flowchart of literature screening.

The selection criteria and literature selection process for this study are shown in Figure 1. Briefly, search formulas for a preliminary search were entered, followed by a review of the publications identified in the initial search, with the following inclusion criteria: (1) Written in English; (2) The types of literature included types are articles, but not letter, comments, reviews, or conference abstract; (3) The publications were from the WoSCC Citation Index Expanded (SCI-E) and Social Sciences Citation Index (SSCI) databases; (4) The search time span was from 2001 (1 January 2001) to 2023 (5 May 2023); (5) The manuscript was based on the theme of the relationship between CRC and gut viruses; and (6) To avoid bias caused by daily database updates, relevant literatures were searched and screened on the same day.

### 2.2. CiteSpace analysis

CiteSpace (6.2.R1) software, a literature visualization application tool created by Prof. Chaomei Chen, is a citation visualization analysis software that is gradually developed under the background of scientometrics and data visualization. Its main function is to convert a large amount of literature data into a visual map.

The parameters of CiteSpace are set as follows: the time slice is chosen from 2001 to 2023, the year of each slice is 1, and the criteria are chosen (g-index, g^2^ ≤ k Σ_i ≤ g_c_i_,k∈ Z^+^,k = 25). The included literature was visualized and analyzed for country/region, institution, reference, and keywords, respectively.

In the generated plots, N denotes the number of network nodes, E denotes the number of connected lines, Density denotes the network density, and Modularity is the evaluation index of network modularity. The larger the Modularity Q value is, the better the clustering obtained by the network. Modularity Q value >0.3 means that the delineated clustering structure is significant. Silhouette value was used to measure the homogeneity of the network. Closer to 1 reflects the higher homogeneity of the network, and above 0.5 means that the clustering structure has reasonableness.

The main measurements are as follows. (1) The analysis of cooperation network among countries/regions, institutes of journals and (2) Cited literature analysis mainly included network diagram, timeline diagram, and references burst. Co-citation means that two (or more literatures) are cited by one or more subsequent literatures at the same time, and the two literatures are said to constitute a co-citation relationship, which is a research method used to measure the degree of relationship between literatures; (3) Keyword analysis mainly included keyword cluster analysis, keyword time zone map and keyword burst analysis. Keyword cluster plots focused on reflecting the structural characteristics between clusters and highlighting their key nodes and important connections. The keyword time zone diagram focused on showing the evolution of high-frequency keywords in terms of the temporal dimension (Zhang et al., 2021). The keyword burst analysis allowed for the exploration of rapidly growing topics in the field (Chen et al., 2014; Luo et al., 2021). A flowchart of the study design is shown in Figure 1.

## 3. Results

### 3.1. Country/region visual analysis

**Figure 2:**
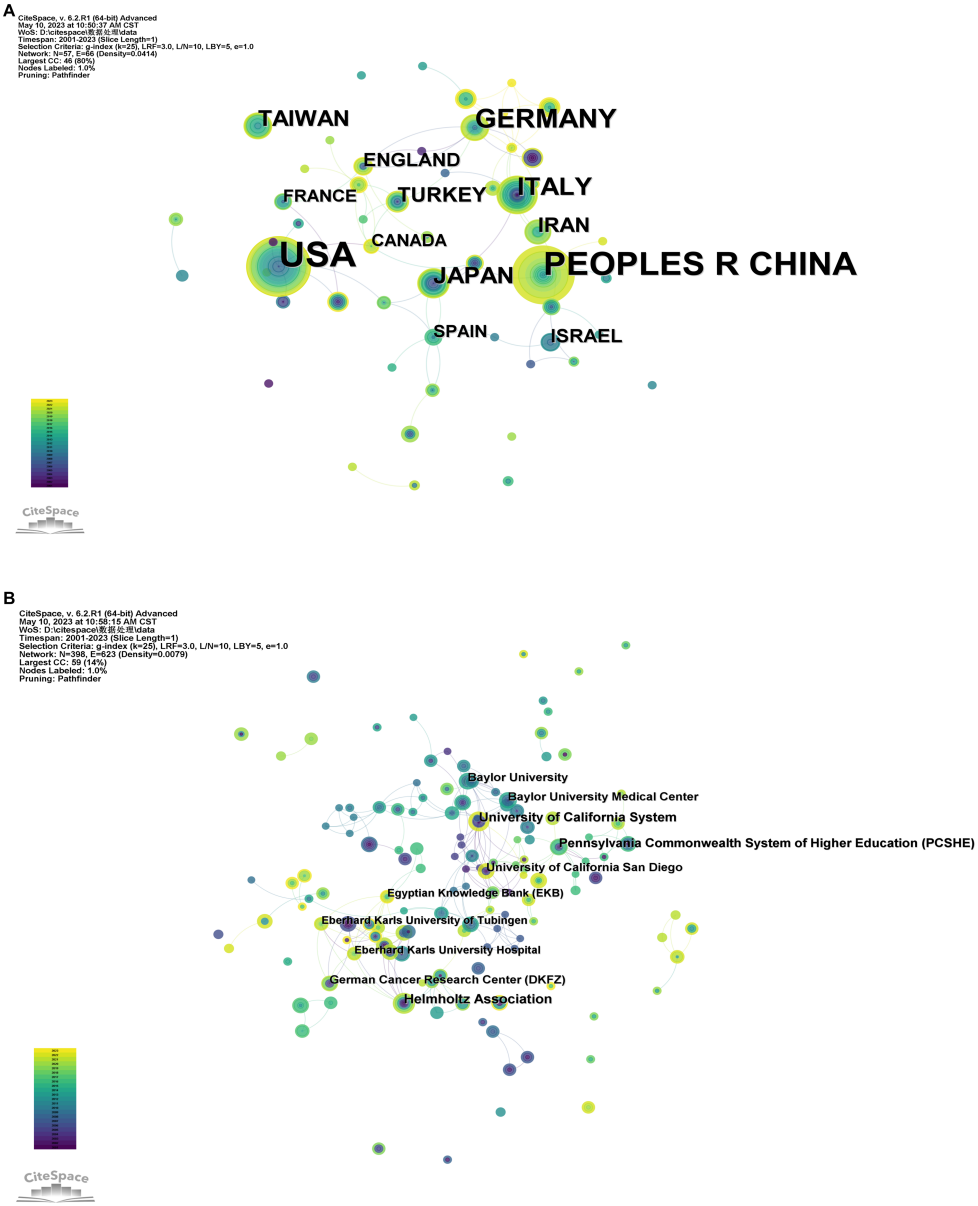
Diagram of the collaborative network of country/regional **(A)** and institutional **(B)** related literature. Nodes represent countries or institutions, and lines connect them. The number of publications is proportional to the size of the node. Connections between nodes represent partnerships. From 2001 to 2023, the color changes from purple to yellow.

The WOS database literature was analyzed, and the CiteSpace software was run with countries as nodes, and the top 3 countries in 57 countries were the United States (107 articles), China (78 articles), and Germany (33 articles). Through data analysis, it was found that China and the United States can be said to be the most core countries in the network, but it can also be seen that the international community’s exchange of relevant research on the relationship between gut virus and CRC is relatively distant. Germany primarily collaborates closely with the United Kingdom, Scotland, Turkey, and Switzerland; China primarily collaborates closely with Australia and Singapore (Figure 2A); The United States collaborates closely with Argentina, Netherlands, South Korea, and Canada.

### 3.2. Visual analytics for research institutions

The Research Institute Collaboration and Co-Emergence Network displays the capacity for interdisciplinary research among research institutions and the collaboration between research institutions. A total of 398 institutions published relevant literature, including the German Helmholtz Association (10 articles), the University of California (9 articles), the Federal Higher Education System of Pennsylvania (8 articles), the University of California, San Diego (7 articles), and Baylor University Medical Center (7 articles) published in the United States (Figure 2B). The most prolific institutions are mainly from the United States and Germany.

### 3.3. Co-cited references and references burst

**Figure 3:**
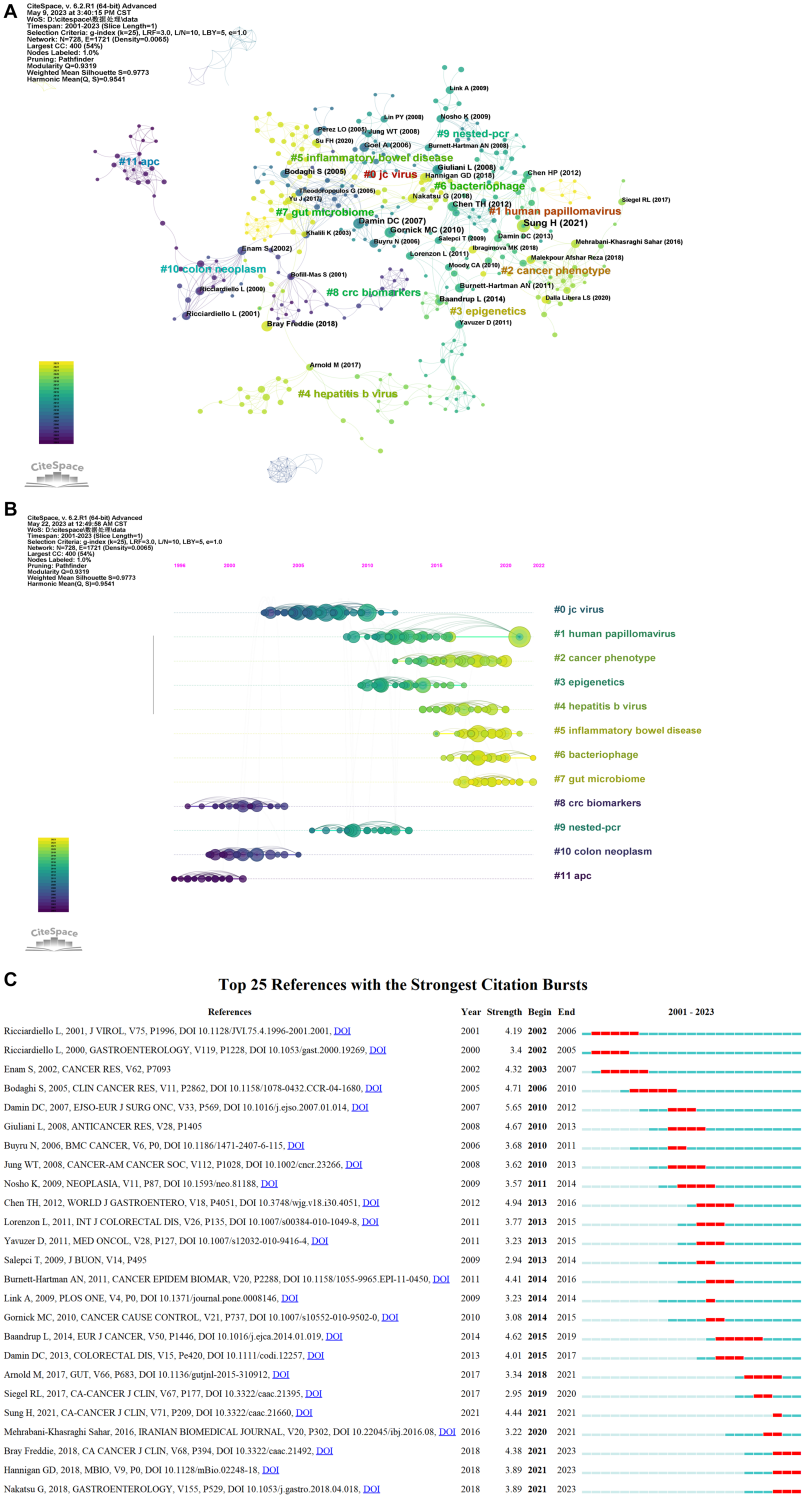
References are co-cited for analysis. **(A)** Cluster view of co-citation of references. **(B)** A timeline view of the co-citation of references. For each cluster, the location of each node represents when the literature was published, and the node size represents the number of citations. **(C)** The 25 most cited references. The blue bars represent the timeline, and the red bars indicate the start and end years of the burst duration.

Among the 356 studies on microbiota and CRC, the visual map generated with “reference” as the node can obtain 728 nodes and 1,721 connected literatures in the network (Figure 3A). Log-likelihood ratio (LLR) can be used to cluster the cited literature and generate 12 groups of cluster labels (#0–11), respectively: “jc virus” (#0), “human papillomavirus” (#1), “cancer phenotype” (#2), “epigenetics” (#3), “hepatitis b virus” (#4), “inflammatory bowel disease” (#5), “bacteriophages” (#6), “gut microbiome” (#7), “crc biomarkers” (#8), “nested-pcr” (#9), “colon neoplasm” (#10) and “APC” (#11). The modularity Q is 0.9319, which indicates that the clustering of the network is reasonable; the silhouette is 0.9773, which indicates that the clustering has good homogeneity.

Based on the co-cited literature, the noun terms in the keywords were extracted, the clustering was annotated, and the timeline diagram of the co-cited literature was obtained (Figure 3B). The distribution from left to right in the center of the node in the Figure represents the year, in which the cited literature was first published and reflected the temporal characteristics and evolution trend of the cited literature (Wang et al., 2021). It can be found that there are some clusters that are currently active, such as “human papillomavirus” (#1), “hepatitis b virus” (#4), “inflammatory bowel disease” (#5), “bacteriophages” (#6), and “gut microbiome” (#7), which represent research hotspots in this field.

In addition, as shown in Figure 3C, the top 25 literatures with the strongest citation bursts based on citation burst values were identified (See details in Table 1). The 25 studies mainly examined the association of specific viruses, including HPV, CMV, and JCV, with CRC and its oncogene mutations, as well as two statistical reports on CRC morbidity and mortality. The first citation burst occurred in 2002 and was published by Ricciardiello et al. (2001). It evaluated the difference in TCR of 8 pairs of JCV isolated from colon cancer tissue and non-tumor epithelium. It was found that the only JCV strain present in the human colon was Mad-1, and only a variant with a single 98 bp sequence was found in cancerous tissue, which may be involved in the development of chromosomal instability (Ricciardiello et al., 2001). The strongest burst in 2010 came from a paper published in 2007 by Damin et al. (2007) which indicated that HPV DNA was detected in colorectal specimens from 60 rectal cancer patients (83.3%), while HPV DNA (*p* < 0.001) was not detected in tissues of non-malignant control groups, and HPV16 was found to be the most common type of virus. The second most frequently cited article is a large international study published in Cancer Cause Control by Gornick et al. that examined 37 human papillomavirus (HPV) in the DNA of paraffin-embedded or frozen tissues in three different populations of CRC patients (279 cases) and normal paracancerous tissues (30 cases) in the United States (73), Israel (106) and Spain (100 cases). All samples were negative for all types of HPV using both the GP5+/GP6+ PCR reverse line blot method and the SPF10 INNO-LiPA method (Gornick et al., 2010). The 3 papers published in 2018 continued to explode, which means that they have received a lot of attention in the field recently. The most recent burst occurred in 2021 and has been continued for 2 years now. There are mainly three literatures as follows. Nakatsu et al. (2018) found a significant increase in the diversity of intestinal bacteriophage communities in CRC patients and altered interactions between bacteriophages and oral bacterial symbionts in fecal samples from CRC patients. In addition to this, Hannigan et al. (2018) proposed that bacteriophage communities are associated with CRC and may influence cancer progression by altering bacterial host communities. It is not difficult to notice that in recent years, the field has paid more attention to the role of bacteriophages in the occurrence and development of CRC.

**Table 1:** Top 25 co-citation representative literature (2001–2023).

Rank	Cited number	Title	Year	Journal	Reference
1	16	Global cancer statistics 2020: GLOBOCAN estimates of incidence and mortality worldwide for 36 cancers in 185 countries	2021	CA Cancer J. Clin.	Sung et al. (2021)
2	11	Evidence for an association of human papillomavirus infection and colorectal cancer	2007	Eur. J. Surg. Onc.	Damin et al. (2007)
3	11	Human papillomavirus is not associated with colorectal cancer in a large international study	2010	Cancer Cause Control	Gornick et al. (2010)
4	10	Human papilloma virus 16 E6 oncoprotein associated with p53 inactivation in colorectal cancer	2012	World J. Gastroenterol.	Chen T. H. et al. (2012)
5	9	Detection of oncogenic DNA viruses in colorectal cancer	2008	Anticancer Res.	Giuliani et al. (2008)
6	9	Colorectal papillomavirus infection in patients with colorectal cancer	2005	Clin. Cancer Res.	Bodaghi et al. (2005)
7	9	The prevalence of human papillomavirus in colorectal adenomas and adenocarcinomas: a systematic review and meta-analysis	2014	Eur. J. Cancer	Baandrup et al. (2014)
8	9	Global cancer statistics 2018: GLOBOCAN estimates of incidence and mortality worldwide for 36 cancers in 185 countries	2018	CA Cancer J. Clin.	Bray et al. (2018)
9	8	Human cytomegalovirus preferentially infects the neoplastic epithelium of colorectal cancer: a quantitative and histological analysis	2012	J. Clin. Virol.	Chen H. P. et al. (2012)
10	8	Association of human polyomavirus JCV with colon cancer: evidence for interaction of viral T-antigen and beta-catenin	2002	Cancer Res.	Enam et al. (2002)
11	8	Mad-1 is the exclusive JC virus strain present in the human colon, and its transcriptional control region has a deleted 98-base-pair sequence in colon cancer tissues	2001	J. Virol.	Ricciardiello et al. (2001)
12	8	Alterations in enteric Virome are associated with colorectal cancer and survival outcomes	2018	Gastroenterology	Nakatsu et al. (2018)
13	8	Association of JC Virus T-antigen expression with the methylator phenotype in sporadic colorectal cancers	2006	Gastroenterology	Goel et al. (2006)
14	8	No evidence for human papillomavirus in the etiology of colorectal polyps	2011	Cancer Epidemiol. Biomarkers Prev.	Burnett-Hartman et al. (2011)
15	8	Diagnostic potential and interactive dynamics of the colorectal cancer Virome	2018	MBio	Hannigan et al. (2018)
16	7	JC virus T-antigen in colorectal cancer is associated with p53 expression and chromosomal instability, independent of CpG island methylator phenotype	2009	Neoplasia	Nosho et al. (2009)
17	7	JC virus T-antigen expression in sporadic adenomatous polyps of the colon	2008	Cancer-Am Cancer Soc.	Jung et al. (2008)
18	7	Global patterns and trends in colorectal cancer incidence and mortality	2017	GUT	Arnold et al. (2017)
19	7	Human papillomavirus infection and colorectal cancer risk: a meta-analysis	2013	Colorectal Dis.	Damin et al. (2013)
20	7	Human papillomavirus and colorectal cancer: evidences and pitfalls of published literature	2011	Int. J. Colorectal Dis.	Lorenzon et al. (2011)
21	6	Demonstration of herpes simplex virus, cytomegalovirus, and Epstein–Barr virus in colorectal cancer	2016	Iranian Biomed. J.	Mehrabani-Khasraghi et al. (2016)
22	6	JC virus DNA sequences are frequently present in the human upper and lower gastrointestinal tract	2000	Gastroenterology	Ricciardiello et al. (2000)
23	6	Evaluation of HPV DNA positivity in colorectal cancer patients in Kerman, Southeast Iran	2018	Asian Pac. J. Cancer Prev.	Malekpour Afshar et al. (2018)
24	6	Investigation of human papillomavirus DNA in colorectal carcinomas and adenomas	2011	Med. Oncol.	Yavuzer et al. (2011)
25	6	Coexistence of K-Ras mutations and HPV infection in colon cancer	2006	BMC Cancer	Buyru et al. (2006)

### 3.4. Keywords visualization

**Figure 4:**
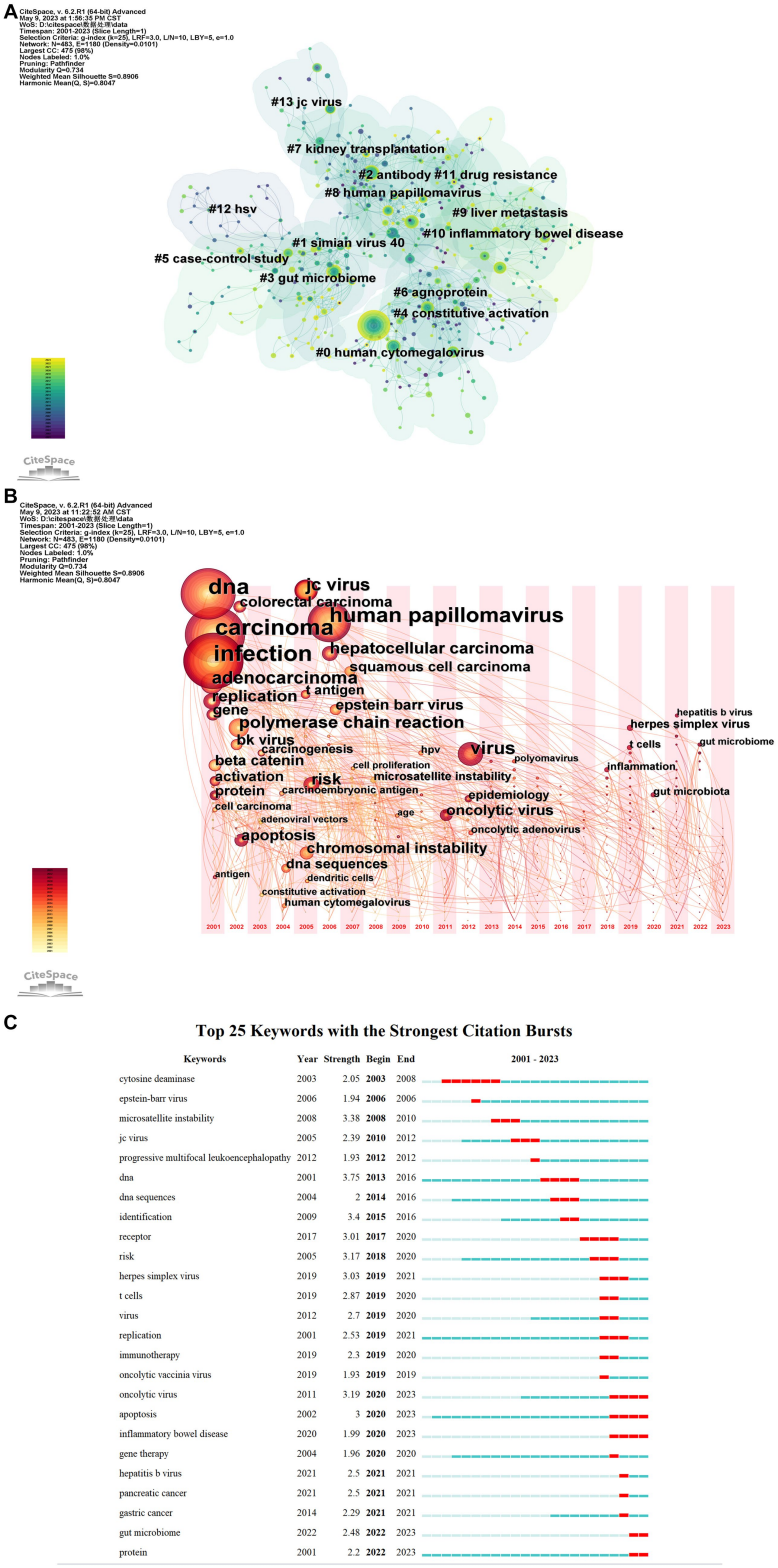
Keyword visual analysis. **(A)** Keyword cluster view: different colors represent different clusters. Each node represents a keyword, and the number on the node represents the cluster to which the keyword belongs. Tags are assigned to the cluster. The smaller the count is, the more keywords there are in the cluster. **(B)** Keyword time zone view: increasing from left to right. Using “1” year as a time slice, keywords that first appeared in the same year are aggregated into the same time zone. **(C)** Show the change of research hotspot over time. The top 25 keywords with the highest citation frequency, with blue lines indicating time intervals and red lines indicating citation burst durations. In the keyword burst analysis, “Begin” and “End” indicate the time of burst. “Intensity” refers to the strength of the burst, which represents the credibility over time.

On the basis of keyword co-occurrence, the keywords were clustered, and a total of 14 clusters were collected, namely “human cytomegalovirus” (#0), “simian virus” (#1), “antibody” (#2), “gut microbiome” (#3), “constitutive activation” (#4), “case–control study” (#5), “agnoprotein” (#6), “kidney transplantation” (#7), “human papillomavirus” (#8), “liver metastasis” (#9), “inflammatory bowel disease” (#10), “drug resistance” (#11), “hsv” (#12), and “jc virus” (#13). From Figure 4A, there are many connections between nodes, which proves that keywords in this field are highly co-presentable. The label numbers in the keyword cluster are inversely proportional to the size of the cluster, and the largest cluster label is human cytomegalovirus (#0), which indicates that the relationship between human cytomegalovirus and CRC has been studied in depth.

As shown in Figure 4B, the research on the relationship between gut virus and CRC in the past decade gradually focused on the study of virus interaction and molecular mechanism. In recent years, herpes simplex virus, hepatitis b virus, t-cells, and the gut microbiome began to appear.

Based on the software’s analysis, a number of keywords with burst have been identified and the top 25 keywords for these burst were selected (Figure 4C), including cytosine deaminase, EBV, microsatellite instability, JCV, progressive multifocal leukoencephalopathy, DNA, DNA sequences, identification, receptor, risk, herpes simplex virus, T cells, Virus, replication, immunotherapy, oncolytic vaccinia virus, oncolytic virus, apoptosis, inflammatory bowel disease, gene therapy, HBV, pancreatic cancer, gastric cancer, gut microbiome, and protein.

### 3.5. Advances in colorectal cancer and gut viruses

This paper summarized the prevalence of HPV, CMV, JCV and other specific virus populations in CRC and how they affect the cellular and microbial microenvironment, which presumably contributes to the promotion of CRC. HPV is a group of circular double-stranded DNA viruses with epitheliophilic properties (Dunne and Park, 2013). Bodaghi et al. (2005) examined cancerous and para-cancerous tissues from 107 CRC samples and found that 36% (38/107) of the samples were infected with HPV16, the most common type of infection. Malekpour Afshar et al. (2018) detected positive HPV DNA in 22.6% (19/84) of CRC samples, with the most prevalent types being HPV types 51 and 56. The study by Giuliani et al. (2008) suggested that HPV and three polyomaviruses, namely SV40, BKV and JCV, were rarely detected simultaneously in the same sample. In addition, there was a correlation between HPV infection and certain clinical features. HPV16 infection was more common in stage I patients than in advanced patients (stages II, III, and IV) (Chen et al., 2012). HPV infection in CRC was different in left site (40%) and right site (20%) (Ambrosio et al., 2023). However, there is heterogeneity in the outcome of HPV infection in CRC. A case–control study by Burnett-Hartman et al. (2011) included a total of 609 colorectal tissue samples from individuals with colorectal adenomas (*n* = 167), hyperplastic polyps (*n* = 87), and polypo-free controls (*n* = 250), none of which had HPV DNA via PCR detection. A meta-analysis revealed that HPV prevalence was 11.2% (294/2630) in adenocarcinomas and 5.1% (21/415) in adenomas in 37 studies (Baandrup et al., 2014). HPV prevalence varies by geographic region, with the highest rates in South America, Asia, and the Middle East (Baandrup et al., 2014).

The possible role of HPV in the development of CRC has been extensively explored. A retrospective study by Lorenzon et al. (2011) found that HPV infection was associated with c-myc amplification, k-ras mutations, and p53 polymorphisms or mutations as potential mechanisms of CRC development. Chen T. H. et al. (2012) discovered that E6 protein was more expressed in HPV16 DNA-positive tumors, and E6 protein may reduce the expression levels of p21 and Mdm2 by inhibiting the transcriptional activity of p53. HPV-related molecular targets may be important mediators of HPV promoting carcinogenesis. The immune landscape and tumor flora of HPV-associated CRC deserve to be deeply explored. According to Ambrosio et al. (2023), HPV-positive tumors exhibited higher abundances and species types and lower abundances in specific genera, the most relevant of which was *Bacteroides*. HPV-negative CRC samples were found in 3 families (*Caulobacteraceae*, *Campylobacteraceae*, and *Xanthomonadaceae*) and 7 genera (such as *Achromobacter*, *Bacteroides*, and *Brevundimonas*), which illustrated that bacteria may influence the interaction between HPV and CRC. Meanwhile, in HPV-associated cancers, tumor environmental immune surveillance is reduced, but lymphocyte cytotoxic responses are enhanced (Ambrosio et al., 2023).

As a herpesvirus group DNA virus, HCMV usually infects humans and sets up a lifelong infection. Most infected people have no clinical symptoms, but under certain conditions, multiple organs and systems can cause serious disease (Yu et al., 2023). Nagel et al. (2023) assessed a CRC incidence of 1.159% (165 cases) in the HCMV group and 2.845% (405 cases) in the control group. HCMV infection was found to be associated with a statistically significant reduction in CRC incidence. Through quantitative and histological analysis, Chen H. P. et al. (2012) found that the detection rate of HCMV DNA in tumor tissues (69/163) was significantly higher than that in neighboring non-tumor tissues (14/163), and the virus copy number of HCMV in tumor tissues was also significantly higher than that in non-tumor tissues. Moreover, HCMV was more likely to infect CRC epithelial cells (Chen and Chan, 2014). HCMV that has cell transformation and possible carcinogenic effects affects a variety of cellular functions, including regulating angiogenesis, promoting cell proliferation, and inhibiting signaling pathways for apoptosis and anticancer immunity (Yu et al., 2023). For example, HCMV is one of the inducers of COX-2, and the overexpression of COX-2 in CRC tissues is associated with poor prognosis (Zhang and Sun, 2002). HCMV-infected cells directly induce angiogenesis by secreting VEGF and other angiogenic factors (Caposio et al., 2011). HCMV infection induces high levels of cyclin, phosphorylated Rb and p53, thus leading to cell cycle arrest (Jault et al., 1995). Therefore, the virus may play a regulatory role in the development of CRC.

JCV, a member of the polyomavirus family, is a double-stranded cyclic DNA virus. JCV infection is very common in both the upper and lower digestive tracts of humans in immunocompetent individuals (Ricciardiello et al., 2000). Jung et al. (2008) explored the role of JCV in precancerous lesions (adenomas) and its relationship with microsatellite instability (MSI). In 61 adenomas carrying JCV sequence, 8% (5/61) were MSI-H, and in 12 adenomatous polyps expressing T-Ag, 8% (1/12) were MSI-H. Furthermore, T-Ag is specifically expressed in the nuclei of these precancerous lesions. Goel et al. (2006) found the JCV T-Ag (JCVT) DNA sequence in 77% (77/100) of CRCs, and the expression of T-Ag in CRC was significantly correlated with genomic instability and methylation of multiple gene promoters, which suggested that JCV may be involved in the occurrence of CRC through multiple mechanisms of genetic and epigenetic instability. Nosho et al. (2009) detected 35% (271/766) JCVT expression in CRCs and found that JCVT was independently associated with p53 expression and chromosomal instability (CIN) in colon cancer through multivariate analysis, but not significantly associated with CPG island methylation phenotype (CIMP). The Wnt/β-Catenin signaling pathway is closely related to the development of CRC (Wong and Pignatelli, 2002). Based on Enam et al. (2002), the T-Ag and the late helper protein Agnoprotein were expressed in CRC. β-catenin appeared in the nucleus and coexisted with T-Ag. β-catenin and T-Ag had a synergistic role in inducing c-myc promoter transcription, which demonstrated that T-Ag may regulate the Wnt signaling pathway through its interaction with β-catenin. Therefore, DNA viruses such as CMV, HPV, and JCV can alter the Wnt/β-catenin pathway in a variety of ways and further affect pathways associated with CRC (Marongiu and Allgayer, 2022).

Besides, phages can regulate gut flora and the immune system (Shuwen and Kefeng, 2022). Single-stranded DNA containing M13 phages is highly immunogenic and can specifically target tumor cell surfaces. CEA specific M13 phage induced tumor regression in a mouse model by specifically binding to CRC cells and activating immune cells and anti-tumor immune responses (Murgas et al., 2018). Combination therapy with M13 phage targeting *Fusobacterium nucleatum* (Fn) and silver nanoparticles can effectively inhibit the development of CRC by remodeling Fn, reducing immunosuppressive cells (MDSCs), and activating antigen-presenting cells (APCs) to remodel the tumor immune microenvironment (Dong et al., 2020). However, it was difficult to establish a single causal link between specific viral infections and CRC, because the gut microbiome is a highly complex ecosystem involving multiple interactions of bacteria, viruses, bacteriophages, and other microorganisms.

## 4. Discussion

Studies have shown that gut microbiota dysfunction is closely related to CRC, so more and more research has focused on the relationship between gut microbiota and CRC over the past 20 years. In this study, relevant studies published in the Web of Science database from 2001 to 2023 were analyzed by manually screening, focusing on the relationship between gut viruses and CRC. CiteSpace V software was used to visually analyze the content of literature and explore research hotspots and frontiers in this field.

The most influential countries and institutions in the field were analyzed. China and the United States are the two main speakers in this field. In our institutional analysis, it was found that 80% of the top 5 output agencies were in the United States, which indicated that the United States institutions issued more publications. Research institutions such as the German Helmholtz Association are relatively mature in this field of research and can be used as important institutions for cooperation and further training.

Co-cited references and key word visualization reflected that research trends and hot topics of gut viruses in the CRC: from the role of specific gut virus (especially HPV) in CRC to the study on bacteriophage and gut microbiome. The timeline view of the reference co-citation analysis can reflect that APC and CRC biomarkers were the first to be developed, while human papillomavirus and bacteriophage were hot topics, which means that research in this field has gradually moved from the representative virus to the study on specific gut viruses in CRC. In terms of keyword frequency, “colorectal cancer” and “expression” are the most prominent keywords. In the past 20 years, with the development of virus metagenomics, the research field between viruses and CRC has developed from detecting the presence of virions to studying the molecular mechanisms of interaction and virus drive. According to the current research on the relationship between gut virus and CRC, there are mainly three aspects: (1) Study on the effect and mechanism of characteristic gut virus on CRC; (2) CRC-associated viral DNA sequences; and (3) Viral therapy can be used on CRC. With the development of metagenomic sequencing technology, gut virus characteristics may be used for CRC diagnosis and prognosis assessment. Chen et al. included 9 published studies on CRC and adenoma (including 1,282 fecal metagenomes) to assemble viral genomes and built a random forest model based on differential bacteriophages identified in CRC patients. It was found that CRC/adenoma and healthy people could be well distinguished (Chen et al., 2022). Yu et al. analyzed viral groups in fecal samples from 74 patients with CRC and 92 control individuals and found a significant increase in bacteriophage diversity in CRC patients. Twenty-two virus genera could be used as markers to distinguish CRC from control (ROC = 0.802) (Nakatsu et al., 2018). In addition, oncolytic viruses (OVs) have been paid more and more attention by scientific research and industry for their ability to replicate specifically within tumor cells and cause tumor cell lysis without affecting normal cells. OVs kill tumor cells through two main principles. One is to stimulate the body’s immune response through the antigen (virus itself and tumor) to activate the body’s natural and secondary immune response and kill the tumor. The second is lysis of tumor cells by selective mass replication in tumor cells for tumor cells lysis and death (Kaufman et al., 2015). Kim et al. (2021) combined OVs therapy with immunocheckpoint inhibitor therapy in a mouse model of colon cancer and found an effective anti-cancer effect. Oncolytic vaccinia virus (VV) showed strong antitumor activity in CRC, and VV had synergistic effects with oxaliplatin or irinotecan (Ottolino-Perry et al., 2015). OVs combined with chemotherapeutic or immunocheckpoint inhibitors is a promising treatment for CRC.

Although the virus is increasingly influencing the pathogenesis of CRC, there is no clear consensus conclusion. Previous studies focused on bacteria (bacteriophages) and eukaryoviruses. To date, other components of the gut virus group have not been studied due to limited understanding of their role in humans and the relatively small virus database. Most reported CRC associations are derived from bacteriophages. Bacteriophages (phages) are the most abundant type of bacterial viruses, which can influence homeostasis through their immunomodulatory and bactericidal effect against bacterial pathogens living in the gut (Gutiérrez and Domingo-Calap, 2020). It has been proposed that bacteriophages might alter the overall balance of the bacteriome by targeting species. Phages promote the expansion of driver bacteria (bacteria that can cause inflammation) and passenger bacteria (bacteria that contribute to cancer), which can lead to the onset and/or progression of colorectal cancer (Hannigan et al., 2018; Khan et al., 2019). In addition, bacteriophages-induced bacteriolysis releases cellular debris into the microenvironment, which can induce inflammation (Tetz and Tetz, 2018).

In the past few years, several human gut virus databases have been published, which have greatly expanded the understanding of the human gut virus genome and provided a wealth of annotated information (Li et al., 2022). The Metagenomic Gut Virus Database (MGV) is a large-scale identification of viral genomes based on 11,810 published human fecal metagenomic sequencing data, obtaining 54,118 vOTUs. These genomes greatly expand the known diversity of DNA viruses in the gut microbiome and improve the understanding of host-virus associations (Nayfach et al., 2021). The Gut Phage Database (GPD) is a broader library of human viruses formed by surveying 28,060 human intestinal metagenomes, containing 142,809 non-redundant bacteriophage genomes. GPD associates bacteriophages with specific bacterial hosts and reveals the global distribution characteristics of human gut virions (Camarillo-Guerrero et al., 2021). These high-quality, large-scale viral and phage genome catalog will improve future virosome studies and enable ecological and evolutionary analysis of human gut virus bacteriophages and studies on their role in CRC.

In this paper, CiteSpace 6.2 R2 software was used to visualize and analyze the literature related to the relationship between gut virus and CRC in the WOS Core Collection database for the past 20 years, but there are still the following limitations. First, the publications we searched were only from SCI-E and SSCI of the WOS database, which may lead to the omission of some publications not included in the database. Second, we only presented English publications in our analysis, which may have omitted some non-English studies. Third, the search terms may have omitted some documents. Finally, the visualization tool is relatively simple. In the future, we can use VOSviewer and Gephi software to provide a more comprehensive and clear theoretical reference for the research in this field.

## 5. Conclusion

CiteSpace bibliometric software was used to analyze and visualize the scientific research productivity and frontiers involved in gut viruses and CRC. China and the United States are the two main speakers in this field. German Helmholtz Association is relatively mature in this field of research. There are mainly three aspects on the relationship between gut virus and CRC as follows: (1) Study on the effect and mechanism of characteristic gut virus on CRC; (2) CRC-associated viral DNA sequences; (3) Viral therapy can be used on CRC. Co-cited references and key word visualization reflected that research trends and hot topics of gut viruses in the CRC: from the role of specific gut virus (especially HPV) in CRC to the study on bacteriophage and gut microbiome. In conclusion, our study provides valuable information for researchers to understand the basic knowledge structure of the field and identify current research hotspots, potential collaborators and future research frontiers.

## Data availability statement

The original contributions presented in the study are included in the article/supplementary material, further inquiries can be directed to the corresponding author.

## Author contributions

QQ and HS: conceived and drafted the manuscript. CJ, WY, and ZJ: wrote the paper. PY and DJ: reviewed and sorted out the literature. CJ: designed and drew figures. All authors contributed to the article and approved the submitted version.
